# Vaccine strategies for cancer prevention in Lynch syndrome: the potential of dendritic cell-based therapy

**DOI:** 10.1007/s10689-026-00559-y

**Published:** 2026-04-22

**Authors:** Romy N. Kuipers, Mark A. J. Gorris, Cristina Bayó, Tom Hofland, Daniel Benítez Ribas, Georgina Flórez Grau, Nicoline Hoogerbrugge, Joaquin Castillo, Francesc Balaguer, Tanya M. Bisseling, Gerty Schreibelt, I. Jolanda M. de Vries

**Affiliations:** 1 Department of Medical BioSciences, Radboud University Medical Center, 6525 GA Nijmegen, The Netherlands; 2 Department of Human Genetics, Radboud University Medical Center, 6525 GA Nijmegen, The Netherlands; 3 Department of Gastroenterology and Hepatology, Radboud University Medical Center, 6525 GA Nijmegen, The Netherlands; 4 Institut d’Investigacions Biomèdiques August Pi i Sunyer (IDIBAPS), Hospital Clínic de Barcelona, 08036 Barcelona, Spain; 5 Department of Gastroenterology, Centro de Investigación Biomédica en Red en Enfermedades Hepáticas y Digestivas (CIBEREHD), Facultat de Medicina i Ciències de la Salud, Hospital Clínic Barcelona, Universitat de Barcelona (UB), Barcelona, Spain; 6 Department of Immunology, Hospital Clinic Barcelona, Barcelona, Spain

**Keywords:** Prevention, Cancer, Vaccines, Lynch syndrome, Hereditary cancer, Dendritic cells

## Abstract

Cancer vaccines offer a promising strategy for cancer prevention, particularly in hereditary cancer syndromes such as Lynch syndrome (LS). LS is characterized by a high lifetime risk of developing various malignancies due to defects in DNA mismatch repair, leading to an accumulation of mutations, particularly frameshift insertion/deletions (indels) within microsatellite loci. These indels create shared tumour-specific neoantigens, which are unique to LS and can be recognized by the immune system and trigger cancer cell killing. Importantly, these neoantigens are also present in precancerous lesions, making LS an ideal target for immune-interception strategies aimed at prevention. Over the years, a variety of vaccine designs targeting different antigens along with a range of delivery platforms have been explored for different types of tumours, each with its own advantages and limitations. In this review, we provide an overview of the key antigens and delivery platforms used in cancer preventive vaccine development for LS, evaluate their limited clinical outcomes to date, and explore the future directions of preventive vaccine immunotherapy. A particular focus is placed on the promising potential of dendritic cell vaccination therapy as a future approach in this field.

## 1. Introduction

Immunotherapy is an active field of research in cancer treatment and prevention [1]. Advances in our understanding of the tumour microenvironment have highlighted the impact of immune-suppressive pathways in cancer progression. This knowledge led to the development of therapies aimed at overcoming these inhibitory signals, with the exceptional successful development of immune checkpoint inhibitors targeting CTLA4 or PD1/PDL1 [2]. Such therapies have demonstrated sustained disease regression in subsets of patients, although not all benefit, and immune-related adverse events remain a challenge [3]. Building on these insights, combinatorial strategies that pair checkpoint inhibition with cancer vaccines have begun to show enhanced efficacy in preclinical and clinical settings [4, 5]. Nevertheless, the overall clinical impact of therapeutic vaccines remains low [6], in part because they are typically administered once disease is already advanced. Preventive vaccination strategies offer a way to circumvent this limitation. Administering vaccines prior to tumour onset, or during early pre-malignant stages, enables the immune system to recognize and control emerging neoplastic cells [7]. In this context, tumours arising in unaffected Lynch Syndrome (LS) carriers, without LS-associated carcinomas, constitute an optimal target for immune preventive cancer vaccination.

### Lynch syndrome and opportunities for vaccine development

LS is one of the most prevalent hereditary cancer syndromes, with an estimated prevalence of about 1 in 300 individuals [8, 9]. Those affected face a lifelong risk of developing multiple types of cancer, with the overall risk reaching up to 70% over their lifetime [8]. LS is an autosomal dominantly inherited syndrome caused by monoallelic germline pathogenic variants (gPV) affecting one of the DNA mismatch repair (MMR) genes: *MLH1*, *MSH2*, *MSH6*, *PMS2* and the 3’ terminal deletion of *EPCAM* [10]. This genetic defect leads to a deficiency in mismatch repair (dMMR), causing rapid accumulation of insertion and deletion mutations (indels), particularly in repetitive DNA sequences known as microsatellites. The high frequency of such mutations is referred to as high microsatellite instability (MSI-H), which is a key hallmark of LS-associated tumours. When these indels occur in coding regions of expressed genes, they result in frameshift mutations that often generate truncated proteins with neoantigens, which are shared across LS patients [11]. While colorectal cancer (CRC) and endometrial cancer (EC) are the most prevalent cancers associated with LS, with risks reaching up to 50% and 45% respectively [8], a broad spectrum of less common cancers are also linked to the condition. These include ovarian, gastric, small bowel, urinary tract, biliary tract, brain, skin (such as sebaceous adenomas, sebaceous carcinomas, and keratoacanthomas), pancreatic cancers [12, 13]. Individuals with LS generally develop cancer at a young age, with the average onset for CRC occurring between 40 and 45 years, and for EC around 50 years [8]. The risk of developing these cancers, as well as the age of onset, can vary depending on the specific gPV present: gPVs in *MLH1* and *MSH2* are typically associated with a higher lifetime cancer risk and an earlier age of onset compared to mutations in *MSH6* and *PMS2* [8].

To mitigate the risks associated with CRC and EC, strict surveillance protocols have been implemented, primarily consisting of regular colonoscopies and transvaginal ultrasound scans aimed at the early detection of (pre)cancerous lesions [8, 14]. While these surveillance strategies have improved outcomes for individuals with LS [15, 16], adherence remains suboptimal. This is likely due to perceived barriers such as discomfort and time consumption, which hinder consistent participation in the recommended screenings [17]. Even with adherence to surveillance colonoscopy, CRC can still develop in patients with LS, as shown in both prospective and retrospective studies [18]. Several explanations have been proposed, including missed lesions due to procedural factors [19–21], accelerated adenoma-to-carcinoma progression [20], and the possibility of an adenoma-free pathway in which CRC arises from submucosal growth or direct neoplastic transformation of dMMR crypt foci that may be difficult to detect endoscopically [22–25]. For other types of cancer associated with LS, surveillance is less effective, largely due to the lower prevalence of these cancers [8]. As a result, no established preventive strategies currently exist for these less common cancer types. Taken together, current surveillance strategies are largely reactive and insufficient to fully prevent cancer development in LS. The persistent lifelong cancer risk continues to impact the quality of life for individuals with LS, contributing to significant anxiety about the potential cancer development. This highlights the urgent need for more proactive strategies, thereby mitigating the risk and reducing the psychological burden on still unaffected LS patients.

In this context, LS represents a particularly suitable model for the development of preventive cancer vaccines. This suitability is supported by several clinical and molecular characteristics of LS. First, individuals with LS carry a high lifetime risk of developing multiple malignancies [26, 27], making them an ideal population for vaccine strategies aimed at inducing sustained immune activation to prevent or delay tumour onset. Second, neoantigens arising in LS-associated tumours have been shown to be highly tumour-specific and immunogenic [28–30], which makes them excellent targets for vaccine therapy. These neoantigens are not subject to central and thymic tolerance and are solely expressed by (pre)cancerous cells [31]. Moreover, the extent of lymphocyte infiltration positively correlated with tumour neoantigen burden [32, 33], highlighting the capacity of LS-tumours to elicit robust T cell responses. Third, several microsatellite loci are highly prone to frameshift indels in dMMR neoplasms, resulting in a high probability that LS patients share the same mutations at these sites [11, 34, 35]. Consequently, neoantigens generated from these recurrently mutated coding microsatellites are commonly shared among multiple LS patients [36, 37]. This shared neoantigen profile presents a unique opportunity to develop broadly applicable cancer vaccines.

The high neoantigen load in LS-associated tumours is associated with substantial lymphocyte infiltration, particularly in tumours of the colon, endometrium, and stomach [38, 39]. Interestingly, these immune responses can also be detected in unaffected LS patients without a personal history of cancer [29], implying premalignant lesions such as colonic adenomas already express neoantigens. These early neoantigens activate CD8 and CD4 T cells and induce proinflammatory cytokines, including tumour necrosis factor (TNF) and interleukin 12 (IL-12) [40, 41]. However, as lesions progress, cancer cells can evade immune surveillance through mutations in antigen presentation genes (e.g., *B2M*,* RFX5*,* CIITA*) or upregulation of immune checkpoint molecules (PD-1, CTLA-4, LAG-3, TIM-3), which suppress T cell activity [42–47]. Together, these findings indicate that neoantigen-targeted vaccination is likely to be most effective in a preventive context, before immune evasion mechanisms are established and tumours acquire more aggressive features.

The purpose of this review is to provide a summary of the current state of LS preventive cancer vaccine development, including description of the different targetable antigens and vaccine delivery platforms. We address key biological and manufacturing aspects of these platforms and discuss past and ongoing clinical trials to identify opportunities for improvement and outline directions for future research.

## 2. Targetable antigens

The development of effective preventive cancer vaccines requires careful optimisation of several key components, among which the selection of an appropriate antigen is fundamental. Selecting the accurate antigen enables the immune system to target and eliminate (pre)cancerous cells while sparing normal tissues, thus preventing autoimmunity. Accurate antigens also induce strong, durable immune memory for rapid responses to emerging tumour cells, whereas poorly chosen antigens may lead to weak T cell activation, tolerance, or off-target responses, reducing preventive efficacy [48, 49]. Selecting true driver antigens is crucial, as their loss reduces tumour survival and they are less prone to immune escape compared to antigens derived from passenger mutations [50]. Earlier studies have explored a range of antigen types, of which their preventive potential varies substantially, both across antigen classes and between tumour entities. In the context of LS, vaccine development predominantly focuses on two major antigen categories: tumour-associated antigens (TAAs) and neoantigens [51], with the latter receiving most attention. An overview of these antigen classes and their defining characteristics is presented in Table 1.

**Table 1:** Targetable antigens for cancer preventive vaccines in Lynch Syndrome

Targetable antigen	Examples	Tumour specificity	Central tolerance	Prevalence among patients	Suitable as preventive vaccine	Risk of auto-immunity
Tumour associated antigens	CEA, MUC1, TBXT	Medium	Yes	Variable	Yes	Present
Shared or common neoantigens	TGFBRII, CASP5, TAF1B, ASTE1, AIM2	High	No	High	Yes	Very low
Personalized neoantigens	Differs per patient	High	No	Very low	No	Very low

CEA carcinoembryonic antigen; MUC1 mucin 1; TBXT T-box transcription factor X. TGFBRII Transforming Growth Factor Beta Receptor 2; CASP5 Caspase 5; TAF1B TATA-Box Binding Protein Associated Factor 1B; ASTE1 Asteroid Homolog 1; AIM2 Absent in melanoma 2

### 2.1 Tumour-associated antigens (TAAs)

Historically, cancer vaccine development initially focused on peptides derived from TAAs [52]. TAAs are protein-derived antigens that tumour cells often constitutively overexpress due to their malignant phenotype. These antigens, which may contribute to cell proliferation and migration, are considered TAAs when they are aberrantly expressed in tumours compared with normal tissues [49, 52]. TAAs can be shared among patients with the same tumour type. In colorectal neoplasia arising in LS, examples include CEA and MUC1 [53]. Notably, some TAAs are also detectable in pre-malignant lesions such as adenomas, although their expression is generally lower than in carcinomas [54, 55]. When expressed at high levels, TAAs may trigger an antitumor immune response by surpassing the threshold for T cell recognition and breaking immunological tolerance.

However, a key limitation of TAA-based immunotherapy is the risk of autoimmunity against normal tissues [56], as these antigens are self-derived. Although TAAs can elicit immune responses, they are typically weakly immunogenic due to their status as self-antigens. Consequently, T cells recognizing TAAs often express low-affinity T cell receptors (TCRs), a feature that has traditionally been associated with limited effector function. Nevertheless, accumulating evidence indicates that lower-affinity T cells can still contribute to tumour control, promote the formation of long-lived memory and provide functional support to higher-affinity T cell populations [57–59]. In addition, T cells specific for TAAs are at risk of deletion via central and peripheral tolerance mechanisms [49], which can further undermine the immune response. Despite the advancement of several TAA-based therapeutic vaccines into clinical trials for various malignancies, robust cytotoxic T cell responses have often not translated into clinical benefit, such as improved disease-free survival or overall survival [60–63]. This may reflect low TCR-antigen affinity, tumour immune evasion through antigen loss, or suppression of cytotoxic activity in the tumour microenvironment.

To date, several clinical trials have been or are currently being conducted to assess safety and immunogenicity of cancer vaccines targeting TAAs in CRC patients [52]. Most studies have focused on therapeutic settings, whereas preventive trials remain scarce. Trials specifically investigating TAA-based vaccines in dMMR or LS patients are particularly scarce, likely because clinical and research efforts have largely shifted toward neoantigen vaccines or immune checkpoint blocking therapies, which exploit the high mutational burden of these tumours. There is one ongoing trial in a preventive setting for unaffected LS patients that uses a viral vector encoding the TAAs CEA, MUC1, and TBXT (NCT05419011). CEA, MUC1, and TBXT are often overexpressed in CRC cells, where they can promote tumour growth, invasion, metastasising capacity, and immune evasion [52, 64, 65]. This phase IIb trial aims to evaluate the safety and immunogenicity of a combination of trivalent adenovirus-5 (Tri-Ad5) vaccines and the IL-15 superagonist nogapendekin alfa (N-803), and to compare the expression of the three TAAs CEA, MUC1, and TBXT in colorectal neoplasms before and after vaccination. The study aims to include 186 LS patients with a prior history of at least one colorectal neoplasm, provided that at least six months have passed since any prior cancer-directed treatment. N-803 is included as it may enhance the migration of CD8 T cells and natural killer cells to B cell follicles, potentially increasing immune responses to the vaccine [66, 67]. Results are expected in 2028.

### 2.2 Neoantigens

The limited clinical results obtained with cancer vaccines based on TAAs urged the development of alternative strategies, particularly those more focused on tumour-specific antigens (TSAs). TSAs are exclusively found on malignant cells and absent from the surface of normal cells. TSAs include mutated neoantigens as well as antigens from oncoviruses [49]. Mutated neoantigens are personalized antigens that arise from tumour-associated nonsynonymous mutations or other genomic alterations, producing altered peptides displayed by HLA molecules on the surface of tumour cells. Because malignant cells accumulate numerous genetic and epigenetic changes that promote their growth, driver mutations are positively selected and maintained, often resulting in altered protein sequences. These altered protein sequences, after processing and presentation by HLA molecules, can lead to the activation of specific T cell responses. These mutation-derived antigens differ from wildtype peptides and can trigger highly specific T cell responses that are not limited by immune tolerance [68]. Their importance is underscored by the observation that tumours with higher mutational burden, producing more neoantigens, tend to respond better to immune checkpoint inhibitors [32, 33, 69].

Advances in next-generation sequencing (NGS) has shifted interest towards further personalized medicine allowing the production of individualized neoantigen-based vaccines. These typically involve obtaining tumour tissue, from which DNA and/or RNA is isolated and sequenced to make predictions of neoantigens that are expressed in that particular patient. In this way, patient-specific vaccines are designed that target highly specific neoantigens derived from dMMR-associated tumours [70].

As previously mentioned, individuals with fully functioning immune systems and high predisposition to cancer would be the best candidates for prophylactic vaccination, as they are likely to mount strong immune responses. Since truly individualized neoantigen vaccines can only be produced in the presence of tumour tissue for neoantigen prediction, these vaccines are difficult to deploy in the prophylactic setting as no (pre)cancerous tissue has developed yet. A more feasible preventive strategy would therefore be to develop broadly applicable cancer vaccines that target recurrent mutations in candidate oncogenes, providing a wider protection through shared neoantigens. In this regard, LS is particularly well suited for preventive strategies as the high frequency of recurrent frameshift mutations produces predictable neoantigens that can be targeted even before malignant lesions develop. For example, a study by Bolivar et al. found that in dMMR colorectal precancerous tissue, up to 100 shared MHC class I neoantigens were identified, with 65 being immunogenic [71]. This suggests that LS could provide a valuable platform for immune-based interventions at an early stage, prior to the development of more advanced malignancies.

Several studies have specifically investigated shared neoepitope-based vaccines in the context of LS and dMMR tumours. Kloor et al., for instance, reported a phase I/IIa trial investigating safety and immunogenicity of a frameshift peptide-based vaccine [72]. This vaccine consisted of three frameshift peptides derived from genes frequently mutated in dMMR tumours: AIM2, TAF1B, and HT001/ASTE1, combined with the adjuvant Montanide ISA-51 VG, a water-in-oil emulsion that enhances the immune response. It was administered subcutaneously to patients with stage III dMMR tumours who had undergone prior curative surgery and standard adjuvant therapy, or to patients with stage IV dMMR tumours who had received prior standard systemic therapy in the first, second, or third line. Patients were excluded if they had received chemotherapy, radiation therapy, or immunotherapy within 4 weeks before study entry. The vaccine was safe and well tolerated. Among 19 evaluable patients, all mounted an immune response against at least one of the vaccine peptides, eliciting both humoral and cellular immunity. However, the clinical efficacy was not assessed, and this study was performed in a therapeutic setting, although Kloor et al. highlighted the vaccine’s potential for use in preventive setting in the future. This potential was further supported by a complementary preclinical study in mice, in which a vaccine comprising of four shared neoantigens from mouse coding mononucleotide repeats was tested [73]. These targets were predicted computationally and validated in vivo in naïve C57BL/6 mice. In VCMsh2 mice, which carry an intestinal-specific Msh2 knockout and spontaneously develop intestinal neoplasms, vaccination significantly reduced intestinal tumour burden, measured as the total weight of all intestinal neoplasms per mouse, and also extended overall survival compared with non-vaccinated mice. Although murine biology differs significantly from that of humans and their MHC molecules do not present human-derived peptides, these findings indicate that frameshift peptide neoantigen vaccination in preventive setting in LS warrants further study.

### 2.3 Challenges in neoantigen targeting

Although targeting neoantigens in preventive vaccines for LS is promising due to their high immunogenic potential and prevalence among individuals, several challenges remain. One major challenge in designing a neoantigen-based vaccine lies in the effective selection of neoantigens. This process typically starts with sequencing the tumour exome and optional RNA sequencing to identify indels in microsatellite regions [74]. Advances in NGS and RNA sequencing now allow large numbers of neoantigens to be predicted directly from DNA and transcriptomic data. However, detecting mutations in microsatellites remains challenging, as it is difficult to distinguish true homopolymer repeat changes from sequencing errors, PCR amplification artefacts, and other sources of noise [75]. To address this problem, Ballhausen et al. proposed an approach of combining DNA sequencing by fragment analysis, focusing on 41 coding microsatellites commonly mutated in dMMR tumours, with in silico fragment length analysis to detect the mutations [36]. This method allowed for precise quantification of frameshift indel mutations while eliminating interference from stutter band artifacts, which may arise from polymerase slippage during DNA amplification and do not reflect true mutations.

After mutation identification, computational analyses are used to prioritize candidate neoantigens for vaccine development. As a first step, HLA typing is performed using whole-exome or RNA sequencing data, for example with tools such as PHLAT or OptiType [76, 77], to determine which HLA molecules are expressed by the individual. Next, mutated peptides are evaluated for their ability to bind to these HLA molecules using in silico prediction tools. Commonly used algorithms include NetMHCpan, MHCflurry, MHCnuggets and VaxRank, which predict peptide-HLA binding affinity based on machine learning models [78–81]. Among these, NetMHCpan is the most frequently used in studies in dMMR and LS-associated tumours [36, 37, 82]. Predicted neoantigens are then ranked based on a limited set of key parameters, such as gene expression, variant allele frequency, predicted MHC binding affinity, and dissimilarity from the corresponding wildtype peptide [83, 84]. Although these computational tools are effective in identifying peptides with high predicted MHC binding affinity, they do not fully account for important biological processes involved in antigen presentation. These include the efficiency of MHC ligand processing, the abundance of the precursor proteins from which neoantigens are derived, proteasomal cleavage efficiency, and peptide transport by the transporter associated with antigen presentation (TAP), all of which influence peptide availability and subsequent loading on MHC molecules [85]. Currently, several integrated pipelines, such as pVAGseq and pVACbind, attempt to incorporate these additional biological processes, however, their predictive performance remains limited due to the scarcity of experimentally validated training data [86]. In addition, most prediction algorithms have historically focused on HLA class I binding, whereas CD4 + T cell responses through HLA class II may be equally relevant for effective tumour control [87, 88]. Accordingly, efforts to develop HLA class II neoantigen predictors have intensified. However, the promiscuous peptide-binding properties of HLA class II molecules complicate accurate prediction, and progress is further limited by the scarcity of experimentally validated datasets for model training [88]. In conclusion, even when a peptide is accurately identified in silico, its immunological relevance ultimately depends on whether it can be effectively processed, presented and recognized in vivo.

This leads to an additional challenge: the substantial diversity of HLA molecules among individuals. Effective cellular immune responses depend on epitope presentation through HLA, with CD4 T cells recognizing HLA class II molecules and CD8 T cells interacting with HLA class I. Because HLA structure determines which epitopes can be bound and displayed, an individual’s HLA haplotype influences the epitope repertoire than can be presented by tumour cells and antigen-presenting cells (APCs) [51]. The high variability across HLA class I and II therefore leads to substantial differences in vaccine efficacy between individuals. This is illustrated by the study of Kloor et al. [72], which evaluated a vaccine consisting of three distinct frameshift peptides in 19 patients with a history of dMMR tumours. As previously mentioned, all participants who completed the full vaccination schedule mounted clear humoral and T cell responses against the peptides. Notably, T cell reactivity was dominated by CD4 responses, whereas CD8 responses were detected in only 9 of 22 individuals. The authors hypothesized and suggested that specific HLA class I genotypes were most likely required to induce CD8 T cell activation and that these genotypes may not have been adequately addressed by the selected frameshift peptides. To overcome this challenge, many trials have restricted their target population to carriers of specific HLA alleles, primarily HLA-A2 specific, as it is the most common allele in the European population, thereby ensuring optimal immunogenicity [89]. However, this narrows coverage of HLA alleles and consequently reduces generalizability. Future research should therefore focus on incorporating different epitopes for multiple neoantigens to expand applicability across diverse HLA backgrounds.

## 3. Vaccine delivery platforms

Once clinically relevant neoantigens capable of eliciting strong and specific immune responses have been identified, the next critical step in vaccine design is selecting an appropriate vector or delivery system to efficiently present these antigens to the target immune cells. In an ideal world, a preventive vaccine should induce a strong and long-lasting cellular and humoral immune response, characterized by high targeting precision and efficient antigen uptake. Equally important is an excellent safety profile, since individuals receiving a preventive vaccine are healthy and should not be exposed to serious adverse effects. Finally, vaccine manufacturing should be feasible and scalable, whether produced as an off-the-shelf formulation or developed through more personalized strategies. Possible delivery platforms for preventive cancer vaccines in LS currently include nucleic acids (DNA and mRNA), viral vectors, synthetic peptides and cellular vaccines, all of which have their own advantages and disadvantages regarding safety, immunogenicity, and manufacturing. An overview of delivery platforms with corresponding published and ongoing clinical trials is shown in Tables 2 and 3.

**Table 2:** Different targetable delivery platforms for cancer vaccines and their applicability in Lynch syndrome prevention. References used [6, 72, 90–94]

	Nucleic acid vaccines			Viral vector vaccines	Peptide vaccines		Cellular vaccines
	Soluble DNA	Soluble mRNA	Nano encapsulated DNA/mRNA	Virus-like particles	Short peptide	Long peptide	Dendritic cells
Mechanism	Delivery of DNA/mRNA/virus into host cell → production of encoded antigen → presentation via HLA molecules of host cell to T cells → immune response				In vivo uptake and processing by APCs → presentation via HLA molecules of APCs to T cells → immune response		Ex-vivo loading of DCs with antigens → re-infusion in patient → presentation via HLA molecules of DC to T cells → immune response
Immunogenicity	Low	Low	Moderate	High	Moderate	Moderate	High
*Advantages*	Can contain high number of antigens Induction of cellular (CD4+ & CD8+) and humoral responses Can co-deliver antigen genes with immune-modulating genes (e.g. cytokines)	Only codes gene of interest Induction of cellular (CD4+ & CD8+) and humoral responses mRNA is self-adjuvant No risk of genome integration	More efficient delivery Protects DNA/mRNA from degradation Endosomal escape possible Inclusion of selective ligands and encapsulated immunogenic compounds can improve targeted delivery	Induction of cellular (CD4+ & CD8+) and humoral responses Durable immune responses without need of adjuvants Targeted delivery to lymph nodes possible Efficient packaging and uptake Can contain high number of antigens	Strong CD8 + response Very specific	Induction of strong cellular (mainly CD4+) and humoral responses Not restricted to bind specific HLAs Can contain several epitopes	Induction of strong cellular (CD4+ & CD8+) and humoral responses Can encode multiple antigens Direct delivery in lymph node possible Long lasting immune response
*Disadvantages*	Inefficient uptake and delivery into cell nucleus Potential risk of integration in genome	Inefficient uptake Highly susceptible to degradation Transient expression of antigens	Nanoencapsulation has poor adjuvant activity Aggregation Rapid clearance Need for booster doses to prolong immune response	Immunity to vector can reduce booster effectiveness Unstable due to lack of viral genome	Limited CD4 + response leading to narrower immune response Require adjuvants Restriction to specific HLAs Inefficient delivery	Limited CD8 + response Less predictable which epitopes will be presented Depends on efficient antigen processing by APCs Require adjuvants Inefficient delivery	Immune response depends on patients DC quality and HLA-type
Safety	High	High	High	Moderate	High	High	Very high
*Explanation*	No serious adverse events Uncomfortable injection	Very rare serious adverse events (myocarditis) or autoimmune events	No serious adverse events	Rare serious events (e.g. VITT) Higher reactogenicity (fever, fatigue) Public trust issue post-COVID	No serious adverse events Very low risk of autoimmunity Induction of CD4 + Tregs may alter immune homeostasis		No serious adverse events Very low risk on autoimmunity Autologous nature reduces immune complications
Manufacturing	Easy	Easy	Moderate	Moderate	Easy	Easy to moderate	Hard
*Explanation*	Manufacturing on large scale possible Easy to purify and store Low-cost		More challenging to produce, especially on large scale	Off-the-shelf and scalable Complex manufacture process High cost	Easy and fast production Low-cost	Harder to synthesize than short peptides Off-the-shelf and scalable	Highly personalized allowing precision medicine Labor-intensive and limited scalability High-cost
Suitable platform for preventive vaccination in LS	No (low immunogenicity, risk of genome integration)	No (low immunogenicity, instable)	Potentially (if natural boosting by emerging adenomas may reduce need for frequent boosters)	Yes (however rare serious adverse events not preferred in preventive setting)	No (inefficient targeting and delivery, no durable response)	Restricted (due to inefficient targeting and delivery and mainly CD4+)	Yes
Current relevant clinical trials in LS	None	None	None	Nous209 (NCT05078866) [95] Nous209 (NCT04041310) TRI-AD5 (NCT05419011)	None	MicOryx(NCT01461148) [72]	DC Vaccination Radboudumc (NCT01885702) [93]DC Vaccination HCB (NCT07163403)

mRNA, messenger RNA; APC, antigen-presenting cell; HLA, human leukocyte antigen; DC, dendritic cell; VITT, Vaccine-Induced Immune Thrombotic Thrombocytopenia; HCB, Hospital Clinic of Barcelona

**Table 3:** Current published/ongoing relevant clinical trials regarding cancer vaccination in patients with Lynch syndrome and/or dMMR tumours

Clinical trial	NCT number	Stage	Setting	Approach	Vaccine composition (number of neoantigens)	Total participants (X^a^ / X^b^)	Key findings/status
Dendritic cell vaccination in patients with Lynch syndrome or colorectal cancer With MSI [93]	NCT01885702	Phase I/II, completed	Preventive and therapeutic	Autologous dendritic cell vaccine	Autologous DC loaded with shared frameshift peptides (*n* = 2)	Therapeutic: 5/3 Preventive: 20/20	Safe, immunogenic (T cell response measurable via biomarker), long-term follow-up (8–10 years) with promising clinical signal
Dendritic cells loaded with frameshift derived neopeptides for cancer prevention in Lynch syndrome (DELAY)	NCT07163403	Phase Ib, not completed	Preventive	Autologous dendritic cell vaccine	Autologous DC loaded with shared frameshift peptides (*n* = 24)	6/20	Active, recruiting.
Vaccination against MSI colorectal cancer [72]	NCT01461148	Phase I/II, completed	Therapeutic	Long peptide vaccine	Long peptides targeting frameshift mutations (*n* = 3)	22/22	Safe, measurable CD4 + T cell & humoral response, one heavily pre-treated patient with stable disease 7 months
Nous-209 genetic vaccine for the treatment of microsatellite unstable solid tumors	NCT04041310	Phase I/II, not completed (since Nov 2022)	Therapeutic	Viral vector vaccine	Adenovirus encoding shared frameshift neoantigens (*n* = 209)	34/115	Active, not recruiting anymore
Cancer preventive vaccine nous-209 for Lynch syndrome patients [95]	NCT05078866	Phase I/II, completed	Preventive	Viral vector vaccine	Adenovirus encoding shared frameshift neoantigens (*n* = 209)	45/45	Safe, neoantigen-specific immune responses after vaccination in 100% (*n* = 37) and durable at 1 year in 85%. Both CD8 + and CD4 + T cell induction.
Testing a combination of vaccines for cancer prevention in Lynch syndrome	NCT05419011	Phase I/II, not completed (since Aug 2023)	Preventive	Viral vector vaccine	Multitargeted recombinant Ad5 + IL-15 superagonist (*n* = 3)	158/186	Active, not recruiting anymore

MSI, microsatellite instable; DC, dendritic cell; Ad5, adenovirus type 5-vector; IL-15, interleukin-15

^a^Total number of participants that was planned to include beforehand

^b^Total number of participants that were eventually included in the study

### 3.1 Nucleic acid vaccines (DNA and mRNA)

Nucleic acid vaccines, including DNA and mRNA platforms, offer an attractive concept: once delivered into host cells, they drive endogenous antigen production capable of eliciting broad CD4 and CD8 immune responses. They are also relatively straightforward to manufacture at scale and have generally favourable safety profiles. However, in their “naked” or soluble form, these vaccines suffer from very poor targeting efficiency and therefore induce only limited immune responses in vivo, largely because the nucleic acids degrade rapidly in systemic circulation [48, 95]. To overcome this, several strategies have been developed to protect and stabilize mRNA and DNA through encapsulation, for example using lipid-based systems or nanoparticle (LNP) formulations, which can be further functionalized with selective ligands to improve targeting [90]. For instance, the well-known COVID-19 vaccines from Pfizer and Moderna utilized LNP formulations to protect the mRNA from degradation [96]. Additional delivery approaches, including microneedle assays, gene gun approaches and in situ electroporation have also been examined to improve transfection [97], without great success. These delivery vehicles often face practical limitations, including aggregation and rapid clearance.

Soluble nucleic acid vaccines are generally unsuitable for preventive cancer vaccination due to their rapid degradation and limited immunogenicity. LNP-based mRNA vaccines, by contrast, have shown strong initial immune responses, as demonstrated in COVID-19 vaccination [96]. However, even with LNP formulations, antigen expression and immune activation remain relatively transient, requiring frequent booster doses to maintain a durable response, which is not ideal in the preventive cancer setting. In the specific case of LS, it is conceivable that the ongoing development of new premalignant lesions such as adenomas with frameshift neoantigens could provide periodic antigen exposure, acting as natural “boosters” to strengthen the immune response and potentially extend protection without the need for repeated vaccine administration. This, however, remains speculative and has not been tested experimentally.

### 3.2 Synthetic peptide vaccines

Synthetic peptides function by delivering peptides into the host, where peptides are taken up in vivo and processed by APCs and subsequently displayed to T cells. This vaccination strategy is attractive because it is relatively safe, inexpensive, rapidly scalable and can be produced in off-the-shelf formats [98]. However, similar to soluble or nanoencapsulated DNA/mRNA vaccines, rapid in vivo degradation remains a major limitation. The use of adjuvants can partially mitigate this issue and enhance immunogenicity.

Synthetic peptide vaccines can be divided into short- and long peptide formats [99]. Short peptides, typically 8–11 amino acids in length, are highly tumour-specific because they are designed to represent precise tumour-associated or tumour-specific epitopes. This allows them to directly bind to MHC class I molecules and efficiently activate CD8 T cells. However, their strict HLA-type restriction limits their ability across diverse patient populations, and they are unable to induce strong CD4 T cell help, which is important for durable immunity [99]. Long peptides, in contrast, are usually 20–35 amino acids long and contain multiple epitopes that require processing by APCs. This enables them to stimulate both CD4 and CD8 T cells and humoral responses, reducing HLA restriction and making them theoretically more suitable for preventive cancer vaccination strategies where a durable immune response is desired. Despite this broader immune response, long peptides often struggle to consistently induce robust CD8 T cell responses, likely due to their dependence on the individual’s APC processing pathways [98, 99]. This limitation was evident in the phase I/II clinical trial conducted by Kloor et al., which tested a long-peptide vaccination contained three frameshift mutation-derived peptides in patients with a history of dMMR tumours [72] (Table 3). The vaccine successfully elicited strong CD4 T cell responses in almost all participants, but less than half of the patients demonstrated a detectable CD8 T cell response, highlighting the challenges in reliably inducing cytotoxic immunity with long-peptide vaccines.

In addition to eliciting effector T cell responses, peptide-based vaccines have been reported to also induce CD4⁺ regulatory T cells (Tregs) [100, 101], which can modulate immune responses and compromise vaccine efficacy. The induction of antigen-specific Tregs could therefore influence the magnitude or functional profile of the vaccine-induced immune response.

Overall, while synthetic peptides vaccines are relatively safe, cost-effective and can be manufactured as off-the-shelf products, their limited ability to generate strong, durable CD4 and CD8 T cells responses reduced their suitability for preventive cancer vaccination strategies in LS and recent trials have focussed more on viral vectors and cellular based approaches.

### 3.3 Viral vector vaccines

Viral vectors have increasingly been explored in recent years as a promising platform for both therapeutic and preventive vaccination due to their inherently immunogenic nature and their ability of efficiently delivering genetic material into host cells [91, 102]. Upon infection, the vector induces endogenous antigen production, which is subsequently processed and presented via HLA molecules to T cells, eliciting broad cellular and humoral responses. Viral vectors enable targeted delivery to lymph nodes, enhancing antigen presentation and promoting stronger, more durable immunity compared with DNA/mRNA or synthetic peptide vaccines. Additionally, this platform allows the simultaneous delivery of a high number of antigens, further increasing the breath of the immune response. Building on this potential, two clinical trials evaluating the Nous-209 vaccine were initiated in 2022 (Table 3). Nous-209 is a viral vector-based vaccine encoding 209 frequently occurring frameshift peptides in patients with dMMR tumours. The first is a phase I/II therapeutic trial investigating the combination of Nous-209 and pembrolizumab, assessing safety, tolerability, and immunogenicity in patients with unresectable or metastatic dMMR cancers (NCT04041310). Peer-reviewed results are not yet available. The second is a phase I/II preventive trial evaluating the safety and immunogenicity of Nous-209 in 45 unaffected LS patients (NCT05078866) [103]. This trial showed that vaccination with Nous-209 was safe with no intervention-related serious adverse events. Neoantigen-specific immune responses after vaccination were detected in all evaluable participants (*n* = 37), of whom 85% maintained durable responses one year after vaccination. Both CD8 + and CD4 + T cell responses were elicited, targeting the encoded frameshift peptides. Another preventive viral vector trial is still ongoing in unaffected LS patients, evaluating the Tri-Ad5 vaccine encoding the TAAs CEA, MUC1, and TBXT in combination with the IL-15 superagonist N-803. This study is assessing safety, immunogenicity, and changes in antigen expression in colorectal neoplasms, with results expected in 2028 (NCT05419011).

Despite their promise, viral vectors are not without limitations. Production is more complex and costly compared with DNA/mRNA and synthetic peptide platforms, although large-scale manufacturing is feasible, which partially mitigates costs. Immune responses against the viral vector itself can reduce vaccine efficacy by neutralizing the vector, thereby limiting both the initial response and effectiveness of booster doses [6]. Moreover, although serious adverse events are rare, viral vector vaccines carry a small but significant risk of severe side effects. Post-COVID, public trust has been impacted by the recognition of very rare events such as vaccine-induced thrombotic thrombocytopenia (VITT) [104, 105], underscoring that a favourable safety profile is essential in preventive settings, where recipients are healthy and any risk of serious adverse events is far less acceptable.

### 3.4 Cellular vaccines (dendritic cell-based)

The final potential platform for cancer vaccination is cell-based. Cellular vaccines are generally classified into several types, including tumour cell-based, DC-based, T cell-based, and natural killer (NK) cell-based approaches [106–108]. In the preventive setting, however, no carcinomas have yet developed, and correspondingly, tumour-specific T cells and NK cells are largely absent. Consequently, with regard to cellular vaccines in preventive cancer vaccination, only DC-based- vaccines are applicable. This platform is distinguished by the use of autologous DCs, which are modified ex vivo and then re-infused to elicit a strong, tumour-specific, broad immune response in vivo. Loading antigens ex vivo enables the incorporation of multiple antigens. Additionally, DC vaccination ensures efficient antigen delivery to the lymph nodes, resulting in a robust and long-lasting immune response. In contrast to viral vectors, DC-based therapies exhibit an excellent safety profile, with only minimal adverse advents consisting of self-limiting flu-like symptoms, fever, and local injection site reactions. Treatment related serious adverse events following DC vaccination are uncommon [109]. Furthermore, the personalized autologous nature of the approach, where the participant’s own DCs are ex vivo loaded with tumour-specific peptides, makes the risk of autoimmunity very rare.

With this in mind, a phase I/II clinical trial was initiated at Radboudumc in 2010 (NCT01885702) to evaluate the safety and immunogenic potential of DCs loaded with frameshift-derived neoantigens. The study enrolled two cohorts: patients with dMMR CRC who had undergone surgical resection within one year before vaccination, and healthy LS individuals harbouring germline dMMR mutations but without any clinical signs of disease (preprint available [93]). All participants received autologous mature DCs pulsed with an HLA-A2-binding short peptide derived from CEA, as well as HLA-A2-binding frameshift-derived neoantigens from caspase-5 and TGF-βRII. As expected, DC vaccination was safe and in nearly all participants (neo)-antigen-specific CD8 T cells were detected. Unlike the few other clinical trials that have investigated preventive vaccination in LS or dMMR, our study is the only one to have addressed clinical outcomes. Notable, we observed that post-vaccination, patients who developed T cells specific for the TGF-βRII neoantigen (39%) did not develop LS-associated neoplasia with TGF-βRII mutations and have remained cancer-free for almost 11 years. Definitive clinical conclusions cannot be drawn due to the small sample size of only 23 patients, however, these findings suggest potential preventive activity and clearly warrant further investigation.

In 2025, a phase Ib clinical trial (NCT07163403) was initiated at the Hospital Clínic of Barcelona to evaluate an autologous DC vaccine loaded with 24 LS–associated frameshift-derived neoantigens. These neoantigens were selected for their predicted high-affinity binding across a wide range of HLA class I and II alleles, ensuring broad immunogenic coverage. The trial is designed as a cancer prevention strategy in unaffected LS carriers. The primary objectives are to assess the vaccine’s safety and to determine its capacity to elicit neoantigen-specific immune responses and is currently in the recruiting phase.

It is important to acknowledge that DC vaccination therapy also has its challenges. The personalized nature of the approach leads to a complex manufacturing process, which is labour-intensive and difficult to scale, resulting in high costs. Additionally, immune response is influenced by both the quality of the participant’s DCs and their HLA-type. The efficiency of antigen presentation and subsequent T cell activation is determined by HLA compatibility [110], which may contribute to variability in immune response.

## 4. Unmet needs and advantages of DC vaccines

Currently, no single platform fully meets all requirements for an ideal cancer vaccine. It is therefore essential to prioritize vaccine characteristics according to the intended context. Therapeutic vaccines may tolerate a degree of adverse effects, given that standard treatments also come with adverse events. In preventive settings, however, minimizing side effects is essential, as participants are healthy. Additionally, inducing a durable and broad immune response without the need for frequent boosters, which could interfere with daily life, is crucial, ideally conferring lifelong protection. Preventive vaccines may also justify higher costs if they reduce the need for subsequent, intensive cancer therapies.

With these considerations in mind, viral vector vaccines and DC vaccines are the two types of vaccines that come closest to the “ideal preventive cancer vaccine”, each with its own set of advantages and disadvantages (Table 2). Both platforms can induce broad and long-lasting immunity, yet differ in susceptibility to tumour immune escape. The Nous-209 vaccine, a viral vector encoding 209 neoantigens, is inherently less prone to escape, as the probability of tumours generating variants not targeted by the vaccine is expected to be low. However, its broad coverage may include neoantigens derived from passenger mutations rather than driver genes, which do not contribute to tumour growth and may dilute immune response without providing additional protective benefit. In contrast, DC vaccines are typically loaded with a smaller set of neoantigens, potentially increasing the risk of escape variants arising over time. However, given the more focused immune response targeting fewer neoantigens, it is possible that this could lead to a more robust and specific immune reaction per neoantigen, potentially enhancing the overall effectiveness of the vaccine.

Viral vector vaccines offer clear manufacturing advantages over DC vaccines, as they can be produced off-the-shelf on large scale, making their deployment more practical. On the other hand, DC vaccination is highly personalized using autologous DCs, which makes large-scale production labour-intensive, time-consuming and costly. Although it is likely that more efficient methods for DC vaccine production will probably be developed in the future, the associated costs are expected to remain higher than for other platforms. In preventive settings, however, higher production costs may be justified if the vaccine demonstrates durable efficacy, as the long-term benefits could outweigh the initial investment.

Safety is particularly important in the preventive setting. As mentioned earlier, safety concerns with viral vectors have emerged, particularly after the rapid development of viral vector-based COVID-19 vaccines, which resulted in some rare but serious complications. DC vaccines, with their excellent safety profile and minimal risk of autoimmunity, may therefore be more suitable for healthy individuals.

In addition to viral vector and DC vaccines, LNP formulated mRNA vaccines could also play a potential role in preventive vaccination for LS. These platforms can theoretically induce broad immune responses, are generally safe, and can accommodate multiple antigens. They also offer off-the-shelf large-scale production advantages compared to DC vaccines. However, no clinical trials have yet evaluated LNP mRNA vaccines in the preventive LS context, likely due to concerns about transient immune responses requiring frequent boosters, which are less desirable in long-term prevention. Nevertheless, given that new adenomas arise throughout life in LS, these neoantigens could serve as natural “boosters,” periodically reinforcing immunity. While speculative, this highlights LNP mRNA vaccines as a potential avenue for future investigation. Nonetheless, considering the combination of durable immune responses, personalized antigen presentation, and excellent safety profile, DC-based vaccines currently appear to be a more attractive platform for preventive vaccination in LS.

## 5. Future directions

Despite the great promise of shared neoantigen-based cancer vaccines compared to vaccines targeting TAAs, several challenges remain before these vaccines can transition from experimental clinical settings to broader clinical application in LS. One of the hurdles is improving neoantigen prediction, especially considering the variability in neoantigen presentation across different HLA molecules. This complicates the design of effective vaccines that can be broadly applicable across diverse patient populations. However, recent advances in single-cell sequencing and bioinformatics are enabling more precise profiling of commonly recurring and shared mutated neoantigens in LS-associated tumours, helping to identify the most immunogenic targets for incorporation in vaccine platforms [83]. An additional challenge in developing preventive vaccines is determining both the optimal number of neoantigens to include and which of these are most likely to elicit a robust immune response. In this context, prioritizing true driver neoantigens is particularly important, as they originate from mutations that are essential for tumour growth. Because the loss of such mutations would reduce tumour survival, these antigens are less likely to be eliminated as an immune-escape mechanism than those derived from passenger mutations. Future research should therefore focus on identifying and selecting the most relevant driver neoantigens to induce strong, tumour-specific durable responses, while minimizing the risk of tumour immune escape by targeting only those antigens that are essential for tumour growth. Another obstacle in vaccine design is ensuring efficient delivery of the vaccine to appropriate sites within the body. In this regard, DCs offer a promising delivery platform due to their ability to naturally migrate to key immune sites, such as lymph nodes where naïve T cells reside, with this process further optimized when injected intranodally for optimal immune activation [111]. Although the labour-intensive and costly nature of DC vaccine production also remains a challenge, the personalized approach offers a significant advantage in terms of safety over other promising delivery platforms, which is essential in preventive settings.

Beyond challenges to antigen selection and vaccine design, the immune context of premalignant lesions may also influence vaccine efficacy. Although immunosuppressive alterations in local microenvironment are most prominent in established malignancies, early premalignant lesions can also exhibit features such as the presence of Tregs, myeloid-derived suppressor cells (MDSCs), and the expression of immune checkpoint molecules including PD-L1 [3]. These mechanisms can dampen cytotoxic T cell activation and may therefore limit vaccine-induced immune responses. In addition, alterations in antigen-presentation pathways, such as B2M mutations, may further facilitate immune escape [3, 112]. In principle, combination strategies incorporating immune checkpoint blockade could help counteract local immunosuppression and enhance vaccine-induced T cell responses. With this in mind, a phase I/II clinical trial is currently carried out evaluating safety and immunogenicity of the frameshift peptide vaccine Nous-209 in combination with pembrolizumab in patients with unresectable or metastatic dMMR cancers in the therapeutic setting (NCT04041310). However, in the preventive setting involving otherwise healthy LS carriers, treatment strategies associated with substantial immune-related toxicity, such as systemic checkpoint blockade [113], are unlikely to be acceptable. Moreover, immunosuppressive mechanisms are typically more dominant in established cancers, whereas premalignant lesions more often retain elements of active immune surveillance. Consequently, the rationale for combining systemic checkpoint blockade with preventive vaccination strategies is considered less compelling.

In parallel with improvements in vaccine design, future research must address the clinical implications of these platforms and delineate the steps needed for their successful implementation. To date, DC-based preventive cancer vaccine studies have focused on enhancing immune responses in individuals carrying the HLA-A*02:01 allele with a limited number of neopeptides. Moving forward, vaccine development will need to shift toward multi-epitope strategies that incorporate peptides capable of binding multiple HLA class I alleles, as well as HLA class II–restricted epitopes, to elicit broader and more durable immune responses. As a notable example of this new direction, the ongoing trial at Hospital Clínic de Barcelona is evaluating a preventive vaccine comprising multiple frameshift-derived neoantigenic peptides that collectively cover a wide range of HLA class I and II alleles. Furthermore, there is a clear need for additional data on vaccine efficacy in relation to clinical outcomes, such as cancer incidence, across larger populations. Consequently, large-scale, placebo-controlled phase III clinical trials are warranted.

Given that preventive vaccination studies often require extended follow-up periods to assess clinical outcomes like cancer incidence, it is also crucial to explore whether biomarkers can be identified to predict vaccine efficacy early on. In the unpublished study with DC vaccination executed by Radboudumc, measuring neoantigen-specific T cell responses is shown to be a potential biomarker for predicting clinical outcomes. This approach is important, as investigating correlations between early immunological responses and later clinical outcomes could enable earlier evaluation of vaccine potential and help optimize trial design.

In conclusion, while shared neoantigen-based vaccines and DC-based platforms show considerable promise in the prevention of tumours, substantial work remains to be done in improving vaccine design, expanding HLA coverage, conducting larger clinical trials, and identifying or validating predictive biomarkers. Continued advancements in these areas will be key to realizing the clinical potential of these vaccines.

## Data Availability

No datasets were generated or analysed during the current study.
